# Proteome Analysis of Rat Hippocampus Following Morphine-induced Amnesia and State-dependent Learning

**Published:** 2015

**Authors:** Saeideh Jafarinejad-Farsangi, Ali Farazmand, Ameneh Rezayof, Niloufar Darbandi

**Affiliations:** a *Department of Cell and Molecular Biology, School of Biology, College of Science, University of Tehran, Tehran, Iran.*; b *Department of Animal Biology, School of Biology and Center of Excellence in Phylogeny of Living Organisms, College of Science, University of Tehran, Tehran, Iran.*; c *Department of Biology, Faculty of Science, Arak University, Arak, Iran.*

**Keywords:** Morphine, Hippocampus, Passive avoidance learning, Protein expression, Proteomics

## Abstract

Morphine’s effects on learning and memory processes are well known to depend on synaptic plasticity in the hippocampus. Whereas the role of the hippocampus in morphine-induced amnesia and state-dependent learning is established, the biochemical and molecular mechanisms underlying these processes are poorly understood. The present study intended to investigate whether administration of morphine can change the expression level of rat hippocampal proteins during learning of a passive avoidance task. A step-through type passive avoidance task was used for the assessment of memory retention. To identify the complex pattern of protein expression induced by morphine, we compared rat hippocampal proteome either in morphine-induced amnesia or in state-dependent learning by two-dimensional gel electerophoresis and combined mass spectrometry (MS and MS/MS). Post-training administration of morphine decreased step-through latency. Pre-test administration of morphine induced state-dependent retrieval of the memory acquired under post-training morphine influence. In the hippocampus, a total of 18 proteins were identified whose MASCOT (Modular Approach to Software Construction Operation and Test) scores were inside 95% confidence level. Of these, five hippocampal proteins altered in morphine-induced amnesia and ten proteins were found to change in the hippocampus of animals that had received post-training and pre-test morphine. These proteins show known functions in cytoskeletal architecture, cell metabolism, neurotransmitter secretion and neuroprotection. The findings indicate that the effect of morphine on memory formation in passive avoidance learning has a morphological correlate on the hippocampal proteome level. In addition, our proteomicscreensuggests that morphine induces memory impairment and state-dependent learning through modulating neuronal plasticity.

## Introduction

Morphine, a potent opiate analgesic drug, has long been used to treat severe pain.Besides relieving pain (1), it can quickly develop tolerance and physical and psychological dependence (2). Furthermore, it has been demonstrated that morphine administration affects learning and memory processes in a dose- and time-dependent manner. For example, acute morphine administration inhibits memory formation in different learning paradigms such as passive avoidance learning (3,4) and spatial learning (5). Our previous studies also showed that post-training administration of morphine induces amnesia in inhibitory avoidance task (3,6,7), while pre-test administration of the same doses of morphine improves morphine-induced amnesia, showing morphine state-dependent learning (7,8). It has been very clearly demonstrated that the newly acquired information in one drug state cannot be recalled or used unless the retrieval is tested in the same drug state (9,10). In spite of several behavioral and pharmacological investigations on morphine state-dependent learning, molecular mechanisms of this phenomenon still await elucidation. 

In general, the mechanisms involved in the modulation of learning and memory processes during exposure to drugs of abuse have long attracted a great deal of attention. Exposure to drugs of abuse such as nicotine, morphine and cocaine has been found to induce neuronal circuit synaptic connectivity in brain areas responsible for their rewarding properties (11). The mesocorticolimbic dopaminergic system which originates from the ventral tegmental area (VTA) and projects to the target sites including the nucleus accumbens, cortex, amygdala and hippocampus, may play a critical role in producing the reinforcing/rewarding effects of the drugs (12). Considering that hippocampal-VTA loop may regulate the entry of information into long-term memory (13), it has been suggested that there is a functional interaction between drug-induced activation of mesocorticolimbic dopaminergic reward system and the induction of hippocampal long-term potentiation (LTP) (14). It should be considered that hippocampal LTP is a critical neural process for memory formation which can be induced in inhibitory avoidance training in rats (15).

Hippocampus as a part of the limbic system is involved in processing of different types of learning (16,17). Electrophysiological studies have shown that there is a relationship between synaptic plasticity in the hippocampal pyramidal neurons and the induction of passive avoidance learning (18,19). According to molecular analyses, synaptic plasticity has two main phases: the short phase which relies on existing proteins (20) and the long one which requires the synthesis of new proteins for long-lasting potentiation of synaptic transmission (21). It is important to note that proteins mediate functional and structural connectivity in neurons during learning and memory processes (22). In order to evaluate the proteins that are responsible for cognitive functions, proteomics has been used as a powerful tool which makes possible simultaneous study of a large number of proteins (23,24). Proteomic studies have shown differential protein expression during hippocampal synaptic plasticity (25,26). For example, it has been shown that the transcription factor cAMP responsive element binding protein (CREB) mediates the conversion of short term synaptic activity to long term plasticity (27).

Differential proteome expression is also associated with morphine dependence (28,29). Molecular analyses have revealed that neuronal energy metabolism, CREB phosphorylation and chaperone proteins level may alter in the hippocampus of morphine-treated rats (28,30). Using passive avoidance learning, Bianchi and colleagues (2009) (31) reported that morphine-induced amnesia increased the activation of hippocampal phospholipase C, which is a signaling effector enzyme in mediating opioid effects (32,33). Considering that morphine is used for pain relief in medicines and that it modulates learning-related plasticity, the aim of the present study was to evaluate expression changes of hippocampal proteins in trained rats treated with saline or morphine in the passive avoidance task by two-dimensional gel electerophoresis and combined mass spectrometry (MS and MS/MS). Therefore, the present work is the first study to determine whether the molecular events required for long-term memory formation of associative hippocampal-dependent tasks can be affected by acute administration of morphine. 

## Experimental

*Animals*

Male Wistar rats (Pasteur Institute; Tehran, Iran) weighing 200-230 g at the time of training were used. The animals were kept in an animal house with a 12-h light/ 12-h dark cycle and controlled temperature (22 ± 2°C). Availability of food and water to animals was *ad libitum*. Animals were kept in an animal room for at least 1 week to adapt to the laboratory conditions before training. All procedures were performed in accordance with institutional guidelines for animal care and use. The Research and Ethics Committee of the college of science at the University of Tehran approved the experimental protocol. 

*Passive avoidance apparatus*

Passive avoidance apparatus for training and testing contained a box of two compartments, one light (white compartment) and the other dark (dark compartment) with the same size (20 × 20 × 30 cm)that were connected to each other with a guillotine door (7 × 9 cm). The floor of the dark compartment was replaced with stainless steel rods (2.5 mm in diameter) at 1cm intervals to produce foot shock. Intermittent electric shocks (50 Hz, 3s, 1mA) were delivered by an insulated stimulator (Borj Sanat, Iran). 

*Training and testing*

*Training*

Training was performed 30 min after habituation of the animals in the experimental room. Each animal was gently placed in the brightly lit compartment of the apparatus; the guillotine door was then opened after 5 s to let the animal enter the dark compartment. The latency with which the animal crossed into the dark compartment was recorded. Any animal waiting more than 100s in the light compartment was eliminated from the study. Once the animal entered the next compartment with all four paws, the door was closed and the animal was taken to its home cage. The trial was repeated after 30 min (acquisition trial), in which a foot shock (50 Hz, 1 mA, 3 s) was immediately delivered to the grid floor of the dark room. The rat was removed from the apparatus and placed temporarily into its home cage. Two minutes later, the procedure was repeated; if the rat did not enter the dark compartment during 120 s, it was recorded as successful acquisition of passive avoidance response. Otherwise, when the rat entered the dark compartment before 120 s, the door was closed and the same shock was applied again. Upon retesting, if the rat acquired passive avoidance successfully, it was removed from the apparatus and put back to its home cage.

*Testing*

Retrieval test was performed 24 hours after training to determine long-term memory. Each animal was gently placed in the light compartment; the door was opened and the step through latency was measured for entering into the dark compartment. Testing session ended when the animal entered the dark compartment or remained in the light compartment for 300 s (criterion for retrieval). During testing session, no electric shock was applied.

*Drugs*

The drug used in the study was morphine sulfate (Temad Co.,Teharan, Iran). Morphine was dissolved in sterile 0.9% saline. Immediately after successful training, morphine was injected subcutaneously (post-training s.c. injection). Control animals received saline subcutaneously (s.c.). 

*Experimental procedure*

*Effect of morphine on memory retrieva lin passive avoidance task*

**Figure 1:**
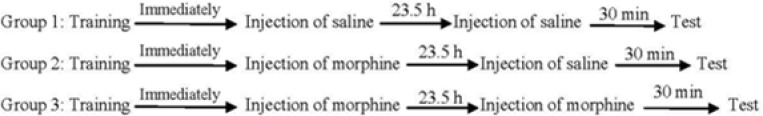
Summery of experimental design in passive avoidance task.

Three experimental groups of eight animals were used in order to evaluate the effects of post-training and/or pre-test administration of morphine on memory retrieval of passive avoidance task. One group of animals received saline (1 mL/Kg, *s.c*.) immediately after training (post-training). On the test day, the animals received saline (1 mL/Kg, *s.c*.) 30 min before the test (pre-test). Another two groups of animals received post-training injection of morphine (7.5 mg/Kg, *s.c*.) and after 23.5 h, they received pre-test injections of saline (1 mL/Kg, *s.c*.) or morphine (7.5 mg/Kg, *s.c*.). The step-through latency was measured 30 min after the injection (Figure 1).

*Hippocampus extraction*

In order to perform hippocampal proteomic analysis, three animals with the best step-through latency score were randomly chosen from each group. Each animal was sacrificed by rapid guillotine decapitation after the test session. The brain was removed from the skull and the hippocampus extracted quickly and easily from rats as described by Chiu and co-workers (2007) (34). The hippocampus was frozen in liquid nitrogen before storing at -80 ˚C. 

*Protein extraction*

Protein extraction was performed as described by Hirano *et al*. (35) with some modifications. Trichloroacetic acid (TCA)/acetone extraction buffer (AEB) was used for protein extraction. TCA-AEB is a suitable solution for protein extraction which denatures proteins and inactivates enzymes (see Table 1). AEB is used to eliminate the interference effect of TCA in isoelectric focusing (IEF). It should be considered that one of the most important considerations in two-dimensional gel electrophoresis (2DE) is the preparation of **protein** samples. All the buffers and detergents should be compatible with IEF. The choice of material for protein extraction requires considerations of salt concentration and protein solubility, because high concentration of salt and protein precipitation can be problematic. Among protocols, TCA-AEB is the most suitable extraction method for reliable IEF. Each hippocampus was homogenized separately with pestle in TCA/AEB, and centrifuged at 15000 rpm, 15 min, at 4 ˚C after incubation for 1 h at -20 ˚C. Lysis buffer (7M Urea, 2M thiourea, 4% CHAPS, 18 mM Tris-HCL (PH 8.0), 50 mM DTT) was added to the pellet and centrifuged at 15000 rpm for 20 min at 10 ˚C. The protein concentration of the supernatant was estimated (20-30 µg/µL) through Bradford method (36) using NanoDrop 2000c Thermo Spectrophotometer.

**Table 1:** Protein extraction solutions

**Material **	**Amount **	**Protein extraction solution**
tricholoroacetic acid (TCA)	10%	TCA-AEB
Merchaptoethanol	0.07%	
Acetone	To needed volume	
Merchaptoethanol	0.07%	AEB
Acetone	To needed volume	
Urea	7M	Lysis buffer
Thiourea	2M	
CHAPS	4% (v/v)	
Tris-HCL (PH 8.0)	18 mM	
Dithioteriol (DTT)	50 mM	
Ultra pure water	To needed volume	

*Two-dimensional gel electrophoresis*

 The first dimension started with Rehydration step in which a Rehydration buffer (8 M urea, 4 % CHAPS, 0.2 % ampholyte pH 3-10, 50 mM DTT ) containing 1mg protein sample was applied onto Non-linear immobilized pH gradient strips (NL IPG, pH 3-10, 17cm). Isoelectric focusing (IEF) was carried out with Protean IEF Cell (BioRad, USA) at 50000 Vh. Before starting the second dimension, Equilibration buffer (6 M Urea, 2% SDS, 50 mM Tris-HCL (pH 8.8), 20% v/v Glycerol) containing 2% DTT (first step) or 2.5% Iodoacetamide (second step) was applied onto focused strips for 20 min. The second dimension was carried out according to Laemmli buffer system (37), using handmade 12% SDS-polyacrylamide gels and run in Protean II xi Cell (bioRad, USA). Each gel was placed separately in bluesilver staining solution for one night (38).

*Image analysis*

Analytical gels were scanned by a Densitometer GS-800 (BioRad) scanner at 300 dpi in tagged image file format (TIFF). Image Master™ 2D Platinum v6.0 software was then used to extract and digitize data from graphical images of scanned gels through detecting, normalizing, matching and comparing protein spots according to their volume percent. Student t-test was performed and spots with more than 1.5 fold changes in vol% selected for identification by mass spectrometry and statistical significance was p<0.05.

*In-gel trypsin digestion of proteins*

Spots were manually cut from 2-DE gels, placed in 96 wells v-shape polypropylene plates and dried completely. The Ettan Spot Handling Workstation (GE Healthcare, UK) was used for automatic in-gel digestion of samples. Each gel plug was soaked in 100 µL of washing solution (50% MeOH, 50 mM NH_4_HCO_3_) to re-swell and was then washed two more times in the same solution. The gel plugs were further washed twice in 75% ACN, before being completely dried. Samples were then re-hydrated by adding freshly prepared trypsin solution (0.5 µg modified porcine trypsin in 25 µL 20 mM NH_4_HCO_3_), and were incubated for 240 min at 37 ^o^C. Peptides were extracted from the gel plugs, by washing twice in 100 µL of 50% ACN, 0.1% TFA and transferred in solution to a fresh 96 well plate, where samples were dried. 

*Matrix-assisted laser desorption ionization time-of-flight mass spectrometry (MALDI-TOF/TOF-MS)*

Tryptic peptides were resuspended in 3 µL of 50% ACN, 0.1%TFA. 0.3 µL of resuspended tryptic peptides were spotted onto a steel Applied Biosystems 192 sample MALDI target plate, and were mixed (while wet) with 0.3 µL of a 90% saturated µ-cyano-4-hydroxycinnamic acid (CHCA) in 50% ACN, 0.1% TFA. The dried samples were analyzed using a MALDI-TOF/TOF MS (4700 Proteomics Analyzers, Applied Biosystems, UK), performing MS analysis and subsequent MS/MS analysis on up to 10 precursor peptides. Each sample was internally calibrated by reference to specific autolytic fragements of trypsin. Mass tolerance settings of 1.2 Da for parent ion and 0.5 Da for fragment ions were applied. Search settings allowed one missed cleavage with trypsin and two modifications (carboxamidomethylation of cysteine and oxidation of methionine). The PMF and MS/MS information were automatically searched against the NCBI non-redundant database using the Mascot search engine (Matrix Science, UK). The Mascot score cut-off value was set to 88 for the statistical confidence limits of 95%.

*Statistical analysis of memory retrieval*

**Figure 2:**
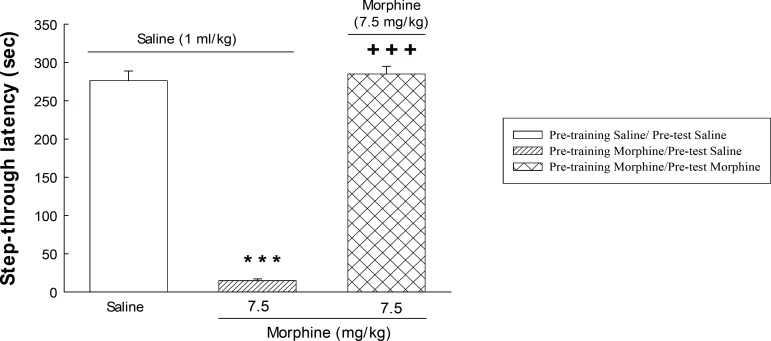
The effect of post-training administration of morphine on step-through latencies. The animals received post-training saline (1 mL/Kg, *s.c*.) or morphine (7.5 mg/Kg) and were tested after 24 h. Each value represents the mean ± S.E.M. of eight rats per group. ***P < 0.001 compared with the saline/saline group.+++*P *< 0.001 compared with the morphine/saline group.

The step-trough latencies as an index of memory retrieval (Figure 2) are expressed as means ± S.E.M. The statistical analysis was performed using one-way analysis of variance (ANOVA). The level of statistical significance was set at p < 0.05. Post-hoc comparison of means was carried out using Tukey test for comparisons. Calculations were performed using the SPSS statistical package(Version 20).

## Results

*Effects of morphine on memory retrieval*

Figure 1 shows the effect of morphine on memory retrieval in passive avoidance task. One-way ANOVA revealed that morphine administration altered memory retrieval [F (2, 21)=269.0, P<0.001]. Further analysis showed that post-training administration of morphine (7.5 mg/Kg, s.c.) impaired memory retrieval, indicating an amnestic effect. Pre-test administration of the opioid restored the impairment induced by post-training injection of the drug. Post hoc analysis showed that maximum restoration was obtained with 7.5 mg/Kg of morphine.

*Identification of differentially expressed proteins*

Comparison of the protein profiles in the control group, the amnesia group and morphine state-dependent learning group showed different protein expression patterns which are mapped in Figure 2. Protein identification was performed by peptide mass fingerprint (PMF) and tandem mass spectrometry (MS/MS) analysis on up to 10 precursor peptides. Detailed characteristics of the identified proteins resulted from MALDI-TOF MS and MASCOT search engine are presented in Table 2. Statistical confidence limits of 95% were applied for protein identification.

**Figure 3:**
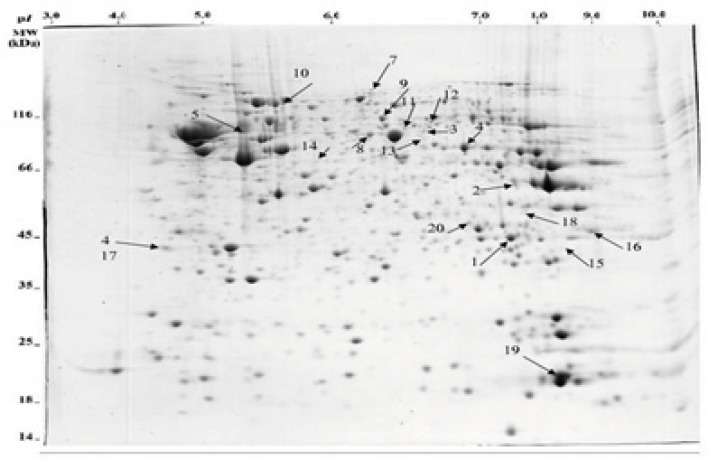
A representation of hippocampus proteome profile following morphine administration. Protein extracts are separated according to their molecular weight at the second dimension using 12% SDS-PAGE. Arrowheads show different expressed proteins.

**Table 2:** Summary of the identified proteins differentially expressed among the three different groups of rats.

**Rank **	**Protein name**	**Acces. No.**	**Protein MW**	**Protein ** ***pI***	**Peptide count**	**Protein score**	**Relative protein content**
1	rCG29914, isoform CRA-b	gi|149049470	31675.8	5.56	13	195	Down-regulation (1.6 folds)
2	Coagulation factor 5/8 type domain protein	gi|150386371	145370.2	8.91	20	88	Down-regulation (1.5 folds)
3	Unnamed protein product **Protein group** Enolase 1, (alpha) isoform1	gi|56107 gi|158186649	47428.3 47440.4	6.16 6.16	13	145	Down-regulation (2 folds)
4	RecName: Full=Synaptosomal-associated protein 25-B; Short=SNAP-B; AltName: Full Synaptosome-associated protein 25.2; Short=SNAP-25.2	gi|82202584	22906.8	4.46	10	102	Up-regulation (2.1 folds)
5	RecName: Full=Tubulin beta-1 chian; AltName: Full=Beta-tubulin class-I **Protein group** Class II beta tubulin isotype Tubulin T beta15 Tubulin, beta2	gi|135446 gi|27227551 gi|224839 gi|4507729	50333.1 50283.1 50361.1 50274.1	4.78 4.82 4.79 4.78	13	145	Up-regulation (2.4 folds)

## Discussion

Morphine which is used for pre and post operative pain management also modulates hippocampal-related learning (7), predominantly through µ-opioid receptors in a time- and dose-dependent manner. Our previous studies had shown that morphine could induce state-dependent learning (StD) which is a dual action of the opiate on learning and memory processes (3). Using passive avoidance learning, our previous results also showed that pre- or post-training administration of morphine induced amnesia via inhibiting the acquisition or consolidation of memory while pre-test administration of the same doses of the drug improved amnesia through facilitating memory retrieval (6,7). Considering that morphine administration alters the proteome profile of the hippocampus, we hypothesized that differential expression of hippocampal proteins may be involved in morphine-induced amnesia and state-dependent learning. It should be considered that neuroproteomics have been widely used to evaluate brain signal complexity and also help diagnose neuronal disorders. For example, the detailed proteome database of hippocampus reported by Fountoulakisand colleagues (2005) may be useful to better understanding of memory formation, and disorders like dementias, recurrent major depression and Cushing’s disease (39). In addition, a few studies, which have focused on neuroadaptive mechanisms associated with morphine dependence, have reported several profound changes in rat brain proteome following chronic treatment (28,29). On the basis of the above findings, in the present study, 2DGE (Figure 3) was used to detect differentially expressed proteins in two groups of animals which showed the dual action of morphine on passive avoidance learning compared with a control group. Three gels per group were prepared and comparison of protein spot patterns of gels across all groups was made by calculating the coefficients of variation of normalized spot volumes. Differentially expressed spots were excised for identification of proteins by combined MALDI-TOF-MS/MS. Finally 18 proteins whose MASCOT scores were inside 95% confidence level were obtained. The detailed properties of the proteins are listed in Table 2. These proteins belong to different functional groups including cytoskeleton, neurotransmitter secretion, energy metabolism and oxidative damage protection. 

**Figure 4:**
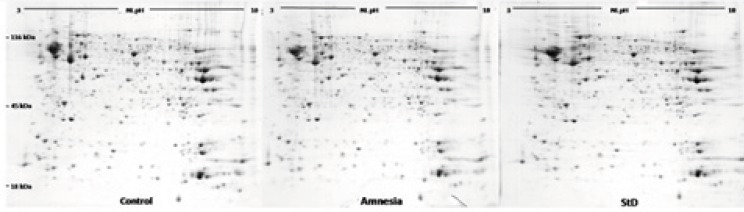
Three representative images of blue silver staining 2DE gels from hippocampal samples of control group, amnesia group and morphine state-dependent learning group. Proteins were separated on 18-cm IPG strips with nonlinear (NL) pH 3–10 in the first dimension and on SDS-polyacrylamide gel (12%) in the second dimension.

The present results showed that five hippocampal proteins altered in morphine-induced amnesia. It was found that post-training administration of morphine increased SANP-25 protein level. SNAP-25 is one of the SNARE proteins which mediate presynaptic vesicular docking processes in exocytosis and neurotransmission release (40,41). This protein exists predominantly in hippocampal glutamatergic synapsis (42), which is the most important excitatory synapses during hippocampal-related learning processes such as passive avoidance learning consolidation (43,44). SNAP-25 has also been shown to regulate neuronal excitability via negatively regulating calcium responsiveness in glutamatergic neurons (42). It has been suggested that down-regulationof SNAP-25 after passive avoidance training (45), increases neuronal excitability required for memory consolidation (46). In our study, down-regulation of SNAP-25 suggest that amnesia, which was induced by post-training administration of morphine, may be partially due to decrease in neuronal excitability. An unnamed protein product which belongs to the group of enolase 1 was down regulated. It is well known that enolase enzymes have an important role in glycosis and glyconeogenesis (47). Inhibitory effect of morphine on glucolytic enzymes has previously been observed (48,49). It is suggested that morphine-induced glucose hypometabolism may result in hippocampal memory impairment (50). It should however be noted that neuron specific enolase has been used as a marker of cognitive disorder (51,52). In addition to glucose metabolism, *α*-enolase also participates in a variety of cellular regulatory processes (53). The obtained results also indicated that post-training administration of morphine up-regulated α-tubulin level. *α*-tubulin is the structural unit of microtubules which mediate the organization of neuronal axons and dendrites in memory-related synaptic plasticity (54). It has been previously shown that the induction of microtubule depolymerization causes amnesia (55,56), Up-regulation of α-tubulin observed in the present study may therefore be due to the depolymerization of microtubules in morphine-induced amnesia. 

Also, in the present study, ten proteins were found to change in the hippocampus of animals that received post-training and pre-test morphine. Interestingly, the majority of changes of hippocampal protein expression have been observed in morphine-induced state-dependent learning. These proteins are functionally divided into the groups of cytoskeleton (Ezrin, Coronin 1A), neurotransmitter secretion (Glul protein, ATPase H transporting lysosomal V1 subunit F) and neuroprotection (peroxiredoxin 5 and rCG55098). Ezrinand coronin 1A are two F-acting binding proteins which exhibit upregulation in morphine-induced StD. Ezrin is one of the ERM (ezrin, radixin and moesin) proteins in peripheral astrocyte processes (PAPs) (57), which mediate positive effects on memory-related synaptic plasticity (58,59). The other cytoskeletal-related protein, coronin 1A, belongs to the type I coronins (60), which mediate the rearrangement of membrane cytoskeleton and may be involved in intracellular membrane transport (61). It has been reported that coronin 1A expression decreases in the hippocampus of aged animals which is indicative of cognitive decline and synaptic loss in the hippocampus (62). Furthermore, glutamine synthetase (Glul protein) is a neurotransmission-related protein which exhibits up-regulationin morphine-induced state-dependency. Glutamine synthetase is an important enzyme which removes excess cerebral glutamate through glutamate–glutamine cycle to avoid neurotoxicity effects of glutamate. Inhibition of Glul protein activity increases extracellular concentration of glutamate (63,64) which results in the impairment of long-term potentiation and cognitive disfunction (65,66). According to data mentioned above, it can be suggested that hippocampal glutamate neurotransmission may have an important role in morphine state-dependent learning. 

On the other hand, a comparative proteome analysis of the hippocampus from two groups of rats that received post-training administration of morphine and pre-test administration of saline (the amnesia group) or morphine (the StD group) showed that hemoglobin *β*-chain protein expression was up-regulated in the hippocampus of the StD group. Hemoglobin gene expression has been reported in different parts of the brain including the hippocampus (67). It should be considered that neuronal hemoglobin chains are sources of hemorphines which bind to opioid receptors and participate in memory retention and retrieval (68). Furthermore, we found that pyridoxine 5’-phosphate oxidase (PNPO) protein level was up-regulatedin morphine dependent leaning group. It is suggested that PNPO up-regulation may increase neuronal excitability and induce long term potentiation in the hippocampus (69,70). Our proteomic approach revealed that morphine administration down-regulated protective enzymes against oxidative damages including Peroxiredoxin 5, carbonyl reductase 1and rCG55098. Moreover, similar to previous studies (48,49), morphine administration altered the expression of proteins involved in energy metabolism including succinate dehydrogenase complex subunit B and an unnamed protein belonging to enolase 1 group. 

The present study offered the first proteome profile of the rat hippocampus in acute morphine administration. Our results revealed differential expression of proteins in morphine-induced amnesia and state-dependent learning. The identified proteins belong to different functional groups including cytoskeleton, neurotransmition, energy metabolism and neuroprotection. Most of the identified proteins including SNAP-25, Glutamine synthetase, *α*-tubulin, ezrin, Coronin 1A and pyridoxine 5’-phosphate oxidase are involved directly or indirectly in synaptic-related processes. Therefore, our proteomic screen suggests that morphine induces memory impairment and state-dependent learning through modulating neuronal plasticity. Our results also showed that a single exposure to morphine can produce prolonged effects on the hippocampal signaling cascades required for long-term memory formation. However, additional assessments are required to further evaluate the outcome of 2 DGE- MALDI-TOF MS method. 
